# Do pregnant women contact their general practitioner? A register-based comparison of healthcare utilisation of pregnant and non-pregnant women in general practice

**DOI:** 10.1186/1471-2296-14-10

**Published:** 2013-01-16

**Authors:** Esther I Feijen-de Jong, Frank Baarveld, Danielle EMC Jansen, Jennie Ursum, Sijmen A Reijneveld, François G Schellevis

**Affiliations:** 1 Department of Midwifery Science, AVAG, Groningen, and the EMGO Institute for Health and Care Research, VU University Medical Center, Amsterdam, 9713 GL, The Netherlands; 2 Department of General Practice, University Medical Center Groningen, PO Box 196, Groningen, 9700 AD, The Netherlands; 3 Department of Health Sciences, and Department of Sociology and Interuniversity Center for Social Science Theory and Methodology (ICS), University Medical Center Groningen, University of Groningen, PO Box 196, Groningen, 9700 AD, The Netherlands; 4 NIVEL (Netherlands Institute for Health Services Research), PO Box 1568, Utrecht, 3500 BN, The Netherlands; 5 Department of Health Sciences, University Medical Center Groningen, PO Box 196, Groningen, 9700 AD, The Netherlands; 6 Department of General Practice and Elderly Care Medicine/EMGO Institute for Health and Care Research, VU University Medical Center, Amsterdam, The Netherlands

**Keywords:** Primary health care, General practitioner, Pregnancy, Health services research, Prenatal care

## Abstract

**Background:**

Midwives and obstetricians are the key providers of care during pregnancy and postpartum. Information about the consultations with a general practitioner (GP) during this period is generally lacking.

The aim of this study is to compare consultation rates, diagnoses and GP management of pregnant women with those of non-pregnant women.

**Methods:**

Data were retrieved from the Netherlands Information Network of General Practice (LINH), a nationally representative register. This register holds longitudinal data on consultations, prescriptions and the referrals of all patients listed at 84 practices in the Netherlands in 2007–2009, including 15,123 pregnant women and 102,564 non-pregnant women in the same age-range (15 to 45 years). We compared consultation rates (including all contacts with the practice), diagnoses (ICPC-1 coded), medication prescriptions (coded according to the Anatomical Therapeutic Chemical classification system), and rate and type of referrals from the start of the pregnancy until six weeks postpartum (336 days).

**Results:**

Pregnant women contacted their GP on average 3.6 times, compared to 2.2 times for non-pregnant women. The most frequently recorded diagnoses for pregnant women were ‘pregnancy’ and ‘cystitis/urinary infection’, and ‘cystitis/urinary infection’ and ‘general disease not otherwise specified’ for non-pregnant women. The mean number of prescribed medications was lower in pregnant women (2.1 against 4.4). For pregnant women, the most frequent referral indication concerned obstetric care, for non-pregnant women this concerned physiotherapy.

**Conclusions:**

GP consultation rates in pregnancy and postpartum shows that GPs are important providers of care for pregnant women. Therefore, the involvement of GPs in collaborative care during pregnancy and postpartum should be reinforced.

## Background

In most industrialised countries midwives and obstetricians are the key providers of care during and shortly after pregnancy, with a recommended number of antenatal visits ranging from six to 15 [1]. This also applies to the Netherlands, where the role of General Practitioners (GPs) as key providers of pregnancy and obstetric care has further declined over recent decades [2-5]. Currently only 2-6% of all practising GPs still provide obstetric care. For most women with uncomplicated pregnancies, primary care midwives provide routine prenatal, intrapartum and postpartum care, and they act as gatekeepers to secondary obstetric care [6,7]. Furthermore, the majority of GP-trainees receive very little theoretical education regarding pregnancy. Despite this, women may still consult GPs as they remain the main providers of routine medical care during pregnancy and postpartum.

Information about the frequency of consultations, diagnoses and management by GPs during pregnancy and postpartum is very scarce. The only available study on this subject was performed by Coco [8]. However, Coco compared rates of additional medical problems unrelated to pregnancy as encountered by GPs and obstetricians while providing prenatal care. He found that GPs made significantly more diagnoses unrelated to pregnancy than obstetricians. However, this study does not provide information about the healthcare utilisation of pregnant women in a healthcare system where midwives and obstetricians are the main maternal healthcare providers.

Evidence on the rates and content of women’s consultations with GPs during pregnancy and postpartum, and the management of these problems by GPs could provide insight into the health and healthcare needs of pregnant women. This evidence could also provide a context for the collaboration and communication between various services and professionals, and the needs for training. Moreover, it provides insight into the role of GPs during pregnancy and the postpartum period.

Therefore, this study aims to compare consultation rates, diagnoses and GPs’ management of pregnant women with those of non-pregnant women in general practice. With this study we can contribute to the understanding of the health status and healthcare needs of pregnant women consulting a GP.

We limited our study to full-term pregnancies, covering the pregnancy period and the first six weeks postpartum.

## Methods

### Study design and setting

We retrieved data from a nationally representative register of GP care: the Netherlands Information Network of General Practice (LINH), including data from 15,123 pregnant women and 102,564 non-pregnant women of the same age range. LINH collects longitudinal data on consultations, prescriptions and referrals [9] of all patients listed in 84 practices in the Netherlands. In the Netherlands every inhabitant has to register with a GP. With respect to the LINH [9] data quality rules, 74 of the 84 participating LINH practices could be used for statistical analyses. None of the participating GPs provided obstetric care.

The LINH register is set up in accordance with national legislation, which does not require medical ethical approval or obtaining informed consent from individual patients. We obtained permission to access the LINH-data from the LINH steering group.

### Participants

Pregnant women were identified in the register using the birthdates of children born in 2007, 2008 and 2009. We selected their mothers and measured their healthcare utilisation within a period starting at 294 days (42 weeks) before the date of birth of their child and ending 42 days (6 weeks) postpartum. Mothers of preterm and stillborn children were excluded. The comparison group of non-pregnant women comprised all non-pregnant women of reproductive age (15–45 years) listed in the same LINH practices. Non-pregnant women were defined as not having given birth to a child and without any indication related to pregnancy in the register. Their healthcare utilisation was measured from July 2006 until June 2010 to create a similar study period. We randomly selected a period of 336 days during these four years for each non-pregnant woman, to get an observation period identical to that of the pregnant women.

### Measures

The primary outcomes in this study were consultation rates, diagnoses and GP management during pregnancy and the postpartum period. *Consultation rates* concerned the number of recorded contacts with the GP practice in the defined 336-day period. *Diagnoses* included the GPs’ assessment of the presented health problem at each contact: diagnoses were coded by the GP according to the International Classification of Primary Care-1 (ICPC-1). The ICPC has been designed to classify symptoms and diagnoses in primary care [10]. It discriminates between symptoms and complaints (ICPC code numbers 01–29), and diagnoses (ICPC code numbers 70–99), further denoted in this paper as ‘symptom diagnoses’ and ‘diagnoses’. Diagnoses were categorised by ICPC chapter, representing the major organ systems. Coding of health problems in the GP practice is incentivised by links in the practice software packages of ICPC codes with procedures enhancing quality of care, e.g. selection of specific patient groups, monitoring medication, or follow-up appointments. *GP management* concerned prescribed medication (automatically coded according to the ATC Anatomical Therapeutic Chemical classification system) and referrals to other care professionals.

We further obtained data on background characteristics, age, socioeconomic status (SES), and level of urbanisation. *SES* concerned the socioeconomic context of the place of residence. This was measured by an existing area score based on mean income, percentage of households with a low income, percentage of inhabitants without a paid job, and percentage of households with only low education, per 4-digit postal code [11-13]. SES was categorised as high (≤ 25th percentile), middle or low (≥ 75th percentile). *Level of urbanisation* concerned the average number of addresses per square km within a radius of one kilometre based on the same 4-digit postal codes. It was categorised as very urban (>2500 addresses/km), intermediate urban/rural (between 500 and 2500 addresses/km) and very rural (<500 addresses/km) [13].

### Analyses

Firstly, we described the background characteristics of our study population. Next, we compared pregnant and non-pregnant women regarding consultation rates, diagnoses, prescribed medication and referrals. If the same diagnosis, prescription or referral was recorded several times, this was counted only once per woman. Stata 11.2 was used for all analyses.

## Results

### Characteristics of the study population

Table 1 shows the characteristics of our study population. There were only small differences between pregnant and non-pregnant women with respect to age and level of urbanisation. More pregnant women lived in both high and low SES areas.

**Table 1:** Characteristics of the study population

		**Pregnant women n = 15,123**	**Non-pregnant women n = 102,564**
Age (mean/SD)		30.6 (4.9)	31.1 (9.6)
SES (%)^a^	High	20.4	14.6
	Middle	46.4	55.0
	Low	32.7	29.8
Urbanisation (%)^b^	Very rural	49.0	48.8
	Intermediate urban/rural	23.3	21.8
	Very urban	27.2	28.9

^a^ Missing: 0.5% (pregnant women), 0.6% (non-pregnant women).

^b^ Missing: 0.5% (pregnant women), 0.5% (non-pregnant women).

### Consultation rates

Table 2 shows the consultation rates of pregnant and non-pregnant women in GPs’ practices. Pregnant women had on average 1.4 more contacts with their GP than non-pregnant women. Of the pregnant women, 35% had no contact with their GP, compared to 50% of the non-pregnant women. Of the women who had contact with their GP, the number of contacts was also higher for pregnant women than for non-pregnant women.

**Table 2:** Number of GP contacts of pregnant and non-pregnant women

	**Pregnant women n = 15,123**	**Non-pregnant women n = 102,564**
	***During pregnancy and postpartum period (336 days)***	***Random period of 336 days***
Number of contacts^a^:		
Median (IQR^b^)	2 (0–5)	0 (0–3)
Mean (SD)	3.6 (4.8)	2.2 (4.1)
Number of women not visiting the GP at all (%)	5,246 (35%)	51,494 (50%)
Number of contacts if visiting:		
Median (IQR^b^)	4 (2–7)	3 (1–6)
Mean (SD)	5.6 (4.9)	4.5 (4.8)

^a^ Differences between the groups are all statistically significant (p < 0.001).

^b^ IQR = inter quartile range.

### Diagnoses and GP management

Table 3 shows the diagnoses recorded by GPs according to the ICPC chapters. These were mostly quite similar for pregnant and non-pregnant women, except for three ICPC chapters that showed differences of more than 10%. First, as would be expected, pregnant women had far more contacts with their GPs for diagnoses related to pregnancy, birth and family planning (ICPC chapter W). Second, pregnant women had fewer contacts with their GPs for musculoskeletal disorders (ICPC chapter L): 17 against 29 per 100 women for non-pregnant women. This difference could not be attributed to a single ICPC code in chapter L. Third, the number of diagnoses for urological problems was higher in pregnant than in non-pregnant women (23 against 9 per 100 women). This was particularly due to higher numbers of cystitis/urinary infections in pregnant women (ICPC code U71, 14 against 6 per 100 women).

**Table 3:** Number of diagnoses by ICPC chapter, per 100 women

	**Pregnant women n = 15,123**	**Non-pregnant women n = 102,564**	**Diff.**
	***During pregnancy and postpartum period (336 days)***	***Random period of 336 days***	
ICPC chapter			
A General	34.7	28.8	5.9
B: Blood, blood forming organs, lymphatic, spleen	4.9	2.8	2.1
D: Digestive	19.5	15.0	4.5
F: Eye	2.5	4.5	−2.1
H: Ear	4.7	6.0	−1.3
K: Cardiovascular	10.9	9.2	1.7
L: Musculoskeletal	17.4	29.5	−12.0
N: Neurological	5.9	7.7	−1.9
P: Psychosocial	7.6	14.9	−7.3
R: Respiratory	23.2	26.1	−2.9
S: Skin	27.3	27.4	−0.1
T: Endocrine/Metabolic and Nutritional	3.2	6.8	−3.6
U: Urological	23.4	8.8	14.6
W: Pregnancy, Childbearing, Family Planning	121.9	10.9^a^	111.0
X: Male Genital	19.9	17.7	2.2
Z: Social Problems	2.7	3.3	−0.6
Mean number of diagnoses per contact (SD)^b^	1.14 (0.4)	1.17 (0.5)	
Mean number of contacts (SD)	3.64 (4.8)	2.24 (4.1)	

^a^ Related to Family Planning codes only.

^b^ More than one diagnosis per contact is possible.

### Most frequently made diagnoses

The ten most frequently recorded symptom diagnoses and diagnoses showed many similarities between pregnant and non-pregnant women; however, there were differences in their ranking. The symptom diagnoses most frequently recorded in contacts with pregnant women were ‘oral contraception’ followed by ‘other localized abdominal pain’, and ‘pregnancy-related vomiting/nausea’ (Table 4). ‘Pregnancy’, the most frequently recorded diagnosis, was recorded in 40.8% of all pregnant women.

**Table 4:** The ten most frequently recorded symptom diagnoses and diagnoses of pregnant and non-pregnant women per 100 women

**Symptom diagnoses**			
**Pregnant women n = 15,123**		**Non-pregnant women n = 102,564**	
***During pregnancy and postpartum period (336 days)***		***Random period of 336 days***	
Contraception oral (W11)	4.3	Contraception oral (W11)	3.8
Abdominal pain localized other (D06)	3.2	Weakness/tiredness general (A04)	2.6
Pregnancy vomiting/nausea (W05)	3.0	Cough (R05)	2.4
Cough (R05)	2.7	Low back symptom/complaint (L03)	2.1
Low back symptom/complaint (L03)	2.6	Abdominal pain localized other (D06)	1.9
Constipation (D12)	2.5	Contraception intrauterine (W12)	1.7
Pregnancy symptom complaint other (W29)	2.3	Foot/toe symptom/complaint (L17)	1.6
Contraception intrauterine (W12)	2.0	Neck symptom/complaint (L01)	1.5
Back symptom/complaint (L02)	1.8	Back symptom/complaint (L02)	1.5
Vaginal discharge (X14)	1.8	Throat symptom/complaint (R21)	1.4
**Diagnoses**			
**Pregnant women n = 15,123**		**Non-pregnant women n = 102,564**	
Pregnancy (W78)	40.8	Cystitis/urinary infection other (U71)	4.0
Cystitis/urinary infection other (U71)	8.7	General disease NOS (A99)	3.6
General disease NOS (A99)	5.9	Upper respiratory infection acute (R74)	3.4
No disease (A97)	5.9	No disease (A97)	2.9
Genital candidiasis female (X72)	5.3	Dermatitis contact/allergic (S88)	2.7
Upper respiratory infection acute (R74)	4.8	Sinusitis acute/chronic (R75)	2.5
Haemorrhoids (K96)	3.2	Allergic rhinitis (R97)	2.2
Puerperal mastitis (W94)	3.2	Dermatophytosis (S74)	2.2
Dermatophytosis (S74)	2.7	Genital candidiasis female (X72)	1.9
Dermatitis contact/allergic (S88)	2.6	Asthma (R96)	1.8

### GP management

Table 5 shows the most frequently recorded medication prescriptions and referrals. Almost half of pregnant women received a prescription, mostly for antibacterials for systemic use (ATC-code J01, 16.4%), followed by prescriptions that could be related to pregnancy symptoms or diagnoses. GPs prescribed medication at least once for 84.2% of non-pregnant women.

**Table 5:** Number of prescriptions and the three most frequently prescribed medications and referrals of pregnant and non-pregnant women

**Prescriptions**			
**Pregnant women n = 15,123**		**Non-pregnant women n = 102,564**	
***During pregnancy and postpartum period (336 days)***		***Random period of 336 days***	
Number of prescriptions:			
Median (IQR)	1(0–3)		2 (1–5)
Mean (SD)	2.1(3.5)		4.4(7.3)
Women without a prescription	47.9%		15.8%
Number of prescriptions in women who received a prescription:			
Median (IQR)	3(1–5)		3 (2–7)
Mean (SD)	4 (4)		5.9(7.9)
**Most frequently prescribed medication per 100 women**			
Antibacterials for systemic use (J01)	16.4	Sex hormones and modulators of the genital system (G03)	20.8
Antianemic preparations (B03)	11.4	Antibacterials for systemic use (J01)	12.2
Gynaecological anti-infectives and antiseptics (G01)	10.8	Anti-inflammatory and antirheumatic products (M01)	10.2
**Most frequent referral to other healthcare professionals per 100 women**			
**Pregnant women n = 15,123**		**Non-pregnant women n = 102,564**	
*During pregnancy and postpartum period (336 days),*		*Random period of 336 days*	
Obstetrician/midwife	5.1	Physiotherapist	1.6
Midwife	3.3	Obstetrician/midwife	1.0
Physiotherapist	2.2	Dermatologist	0.9

Pregnant women were most frequently referred to the obstetrician/midwife in secondary care (7%), followed by midwives in primary care (4%). Non-pregnant women were most frequently referred to a physiotherapist (1.6%), followed by medical specialists.

## Discussion

### Key results

Pregnant women contacted their GPs an average of 3.6 times during pregnancy and postpartum, in addition to the care provided by midwives or obstetricians. They had on average 1.4 more contacts with their GPs than non-pregnant women. The diagnoses made by GPs for pregnant women and non-pregnant women were quite similar. Most of the diagnoses recorded for pregnant women were related to pregnancy problems. However, differences appeared regarding urological problems and musculoskeletal problems. Urological problems were more often recorded with pregnant women, whereas non-pregnant women had musculoskeletal problems more often. The number of prescribed medications was much lower in pregnant women than non-pregnant women. Medication prescribed during pregnancy and postpartum mainly concerned pregnancy-related medication. Finally, pregnant women were most frequently referred to obstetrical healthcare professionals, whereas non-pregnant women were most frequently referred to physiotherapists.

### Interpretation

This is the first study to examine the consultation rates, diagnoses and management of pregnant women in general practice. We found that, in addition to the maternal care provided by midwives and obstetricians, pregnant women have more contacts with their GP during pregnancy and the postpartum period compared to non-pregnant women. The excessive number of contacts related to pregnancy, birth and family planning can explain this difference. Pregnant women might be more worried about their health, resulting in a lower threshold for consulting their GP. Furthermore, pregnancy is a special period in which more health problems occur and extra care is required [14]. However, we do not know why pregnant women choose to consult a GP instead of their obstetric care providers. This is remarkable because GPs in the Netherlands are not the key professionals providing obstetric care. Maybe, pregnant women are more familiar with their GP as compared to their midwife or obstetrician, or women do not have the knowledge to decide whether a symptom is related to pregnancy. Regarding obstetricians, difficulties in getting an appointment may also play a role. Finally, regarding midwives, some pregnant women may primarily contact their GP if they expect that they will need medication, which cannot be prescribed by their midwife.

Our finding that pregnant women consult their GPs frequently for problems unrelated to pregnancy is in agreement with Coco’s study [8] that showed that GPs who provide prenatal care also address non-obstetrical problems frequently. Detailed comparisons of our results with those of Coco are not possible due to different classification systems (ICD-9 vs. ICPC-1), a different study setting (family physicians providing prenatal care vs. GPs not providing prenatal care), and different study variables (exclusion of pregnancy related diagnoses vs. inclusion of all diagnoses).

‘Pregnancy’ was recorded for 41% of all pregnant women. This could be interpreted as not every GP recording pregnancy in the electronic medical record, even though they may know about it. For instance, in the UK the percentage of women visiting a GP as the first professional seen during pregnancy is 82.5% [4]. Obviously, every GP needs to know about a pregnancy and to have this recorded in the medical record: this information is indispensable when problems arise or medication has to be prescribed. On the other hand, midwives and obstetricians should inform GPs about their client’s pregnancy.

We found more diagnoses of cystitis in pregnant women, which confirms that cystitis occurs more frequently during pregnancy. The lower number of musculoskeletal problems of pregnant women presented to their GP could be explained by the commonly held belief among women that musculoskeletal symptoms are to a certain extent ‘normal’ during pregnancy and postpartum and that they need no special attention. In addition, GPs might have coded musculoskeletal problems in pregnant women under the pregnancy-related problems chapter of the ICPC (W); for instance code W29 ‘Pregnancy symptom/complaint other’ instead of musculoskeletal problems (ICPC chapter L).

Medication was less often prescribed to pregnant than to non-pregnant women, which reflects the justified reluctance to prescribe medication during pregnancy because of the potential teratogenic effects of medication use during pregnancy.

Finally, pregnant women were more often referred to other healthcare professionals compared to non-pregnant women. Although musculoskeletal problems were less frequently recorded in pregnant women, they were relatively frequently referred to a physiotherapist. Referring pregnant women to physiotherapists could replace a drug prescription.

### Strengths and limitations of the study

A major strength of our study is the use of a very large and nationally representative dataset of contacts, prescriptions and referrals for both pregnant and non-pregnant women.

This study also has some limitations. First, the recording of data is not always complete, despite completeness being quite high. However, the amount of missing data did not differ between pregnant and non-pregnant women, making an impact on our findings unlikely. Evidently, regarding the diagnosis ‘pregnancy’, many more cases were missed (59%). We do not think that such an under-registration also holds for other diagnoses, which the GP considers to be less self-evident. Moreover, we had no data on other potentially relevant background characteristics such as ethnicity, making it impossible to assess ethnic subgroups. Third, a limitation could be that the pregnant women group had a higher proportion of women belonging to the low and high SES group than non-pregnant women. However, it is unlikely that we missed any women, as all Dutch inhabitants are listed at a general practice. This finding probably reflects that women are less likely to become pregnant in middle SES areas.

### Implications

Our findings have implications for education, research and daily care. The apparently important role of GPs for pregnant women during their pregnancy should result in the training of GPs to recognise and manage health problems during pregnancy and obstetric emergencies.

Second, future research is needed to get more insight into the reasons pregnant women to seek GP care. A better understanding of the pregnant women’s perspective will enable all healthcare professionals involved to respond more appropriately to the needs of pregnant women.

Third, GPs have to participate in the obstetrical healthcare provider team, and may provide shared care as already occurs in some countries like Ireland [15].

Collaboration and sharing of relevant information should be organised. An integrated digital environment can facilitate this communication. Software tools could be helpful for appropriately recording pregnancy in the electronic medical record, e.g. by prompting this on the occasion of a pregnancy test or a referral to a midwife or obstetrician.

## Conclusions

Even where midwives and obstetricians are the key professionals in obstetrical healthcare, women consider their GPs as important care providers during pregnancy and postpartum. This indicates the need to involve GPs in the collaborative pregnancy and postpartum care.

## Competing interests

The authors declare that they have no competing interests.

## Authors’ contributions

EF-J had full access to all of the data in the study and takes responsibility for the integrity of the data and the accuracy of the data analysis. EF-J, DJ, FB, FS, and SR conceived and designed the study; EF-J, FS, and JU acquired the data; EF-J, and JU analysed the data; EF-J, FB, DJ, JU, SR and FS, interpreted the data; and EF-J, FB, DJ, and FS drafted the manuscript. DJ, FB, DJ, JU, SR, and FS critically revised the manuscript for important intellectual content. All authors approved the final version for publication.

## Authors’ information

EF-J: Midwife, lecturer and PhD student, Midwifery Academy Amsterdam-Groningen, Groningen, The Netherlands. FB: General Practitioner, Groningen, the Netherlands. DJ: Assistant Professor at the Department of Health Sciences, the Department of Sociology and Interuniversity Center for Social Science Theory and Methodology, University of Groningen, the Netherlands. JU: Researcher at the Netherlands Institute for Health Services Research, Utrecht, the Netherlands. SR: Professor of Community Medicine at the Department of Health Sciences, University Medical Center Groningen, the Netherlands. FS: Professor of General Practice at the Department of General Practice and Elderly Care Medicine/EMGO Institute for Health and Care Research, VU University Medical Center, Amsterdam, the Netherlands.

## Pre-publication history

The pre-publication history for this paper can be accessed here:

http://www.biomedcentral.com/1471-2296/14/10/prepub
